# HMM-ModE: implementation, benchmarking and validation with HMMER3

**DOI:** 10.1186/1756-0500-7-483

**Published:** 2014-07-30

**Authors:** Swati Sinha, Andrew Michael Lynn

**Affiliations:** 1 School of Computational and Integrative Sciences, Jawaharlal Nehru University, New Delhi 110067, India

**Keywords:** HMM profile, Emission probability, Annotation, GPCRs

## Abstract

**Background:**

HMM-ModE is a computational method that generates family specific profile HMMs using negative training sequences. The method optimizes the discrimination threshold using 10 fold cross validation and modifies the emission probabilities of profiles to reduce common fold based signals shared with other sub-families. The protocol depends on the program HMMER for HMM profile building and sequence database searching. The recent release of HMMER3 has improved database search speed by several orders of magnitude, allowing for the large scale deployment of the method in sequence annotation projects. We have rewritten our existing scripts both at the level of parsing the HMM profiles and modifying emission probabilities to upgrade HMM-ModE using HMMER3 that takes advantage of its probabilistic inference with high computational speed. The method is benchmarked and tested on GPCR dataset as an accurate and fast method for functional annotation.

**Results:**

The implementation of this method, which now works with HMMER3, is benchmarked with the earlier version of HMMER, to show that the effect of local-local alignments is marked only in the case of profiles containing a large number of discontinuous match states. The method is tested on a gold standard set of families and we have reported a significant reduction in the number of false positive hits over the default HMM profiles. When implemented on GPCR sequences, the results showed an improvement in the accuracy of classification compared with other methods used to classify the familyat different levels of their classification hierarchy.

**Conclusions:**

The present findings show that the new version of HMM-ModE is a highly specific method used to differentiate between fold (superfamily) and function (family) specific signals, which helps in the functional annotation of protein sequences. The use of modified profile HMMs of GPCR sequences provides a simple yet highly specific method for classification of the family, being able to predict the sub-family specific sequences with high accuracy even though sequences share common physicochemical characteristics between sub-families.

## Background

Heuristic methods like BLAST [1] and FASTA [2] are commonly employed for the task of assigning function to a protein sequence on the basis of sequence similarity. In many cases, where a close homolog with known sequence and structure is not known, the sequence-sequence comparison methods show poor sensitivity. Profile Hidden Markov Model (HMM) like HMMER [3] and SAM [4] provide increased sensitivity in detecting remote homologs as sequence profiles are a better representation of a set of homologous sequences than a single sequence. Profile HMMs however have poor specificity in case of protein families with closely related function because of the high probability of selecting sequences from other sub families based on fold signals common to the family. The Pfam database [5] uses curated thresholds as an additional aid to the E-value: a Trusted Cutoff (TC), a Noise Cutoff (NC) and a Gathering threshold (GA), where TC > GA > NC. These criteria do not hold uniformity when applied to pre-classified positive and negative training sequence data because there may be cases in which negative sequences have higher scores than positive sequences. Earlier work, though not in wide spread use, have attempted to minimize the effect of non- discriminating residues for fold and function level classification through varied concepts like using negative examples for training HMMs, through the modification of emission [6] and transition probabilities [7] and through using positional entropy [8] to classify sequences both at fold and function level. HMM-ModE [9] is a method that generates family specific profile HMMs, through HMMER, by optimizing the discrimination threshold using the mode of average MCC (Mathews correlation coefficient) distribution from 10-fold cross validation and modifying the emission probabilities using negative training sequences. The protocol is much faster in training because only the sequences selected as false positives by the subfamily HMM, are used to modify model parameters and optimize the discrimination threshold. It provides a significant improvement over the existing methods for classification of fold and function specific signals. Another reason for the limited use of profile HMMs in primary annotation of functions is that the algorithms for database searching were significantly slower than heuristic local alignment methods. Recently, HMMER has been upgraded to a new version which improves database search speed by several orders of magnitude [10]. We have implemented our method to use the recent release of HMMER. This new version of HMM-ModE with HMMER3 will be useful for the large scale deployment in sequence annotation projects. In this work, the performance of the method is benchmarked using both HMMER2 and HMMER3, and validated on a set of pre-classified enzyme superfamilies which are clustered according to specific sequence, structure and functional criteria to be used as a gold standard in family and superfamily clustering methods [11]. In addition, we have also compared all the results reported in this manuscript with the earlier version of the method.

The separation of the fold and function specific signals is important and is a powerful way for classification when the sequences are classified into families and subfamilies as evident from GPCRs, protein kinase and Enzyme classification. Another similar method and resource for automated sub-family classification and identification was developed earlier through a three stage process, estimating a functional hierarchy for each protein family and sub families; using Hidden Markov Models to model both the family-defining and sub-family defining signatures; and using sub-family HMMs to assign novel sequences to functional subtypes [12]. Our focus has been to improve the specificity for any dataset which is classified in a hierarchical fashion. One of the examples for such dataset is G protein coupled receptors (GPCRs).

GPCRs constitute a large family of integral membrane proteins. They form a unique modular system for signal transduction thereby allowing transmission of various signals across and between cells. The name GPCR reflects its involvement in the process of receptor signalling in the cellular environment via GTP binding proteins. They are known to mediate a variety of cellular and physiological signals and are also known as seven transmembrane (7TM) receptors [13]. Found only in eukaryotes, they are an important family of proteins both at the physiological level, where they mediate functions like signal transduction, and at the pharmacological level, serving as important drug targets. Therefore, much of the effort at the research level is now focused on the development of methods for accurate classification of GPCRs. However, since GPCRs are known to have functional as well as sequential diversity hence their classification is a daunting task.

In recent times, one of the most comprehensive and widely used classifications for the GPCR families is provided by GPCRDB [14]. The resource classifies GPCRs into five classes: Class A is the Rhodopsin like, Class B is Secretin like, and Class C is the Metabotropic glutamate/pheromone, Vomeronasal receptors (V1R and V3R) and Taste receptors (T2R). These classes are further classified into sub-families and sub- subfamilies based on the function of GPCR protein and the substrate specificity.

As expected from the growing interest of both academic and industrial researchers, several methods have been proposed for the prediction and classification of GPCRs. Earlier methods made use of covariant discriminant algorithm [15] and bagging classification tree [16] to classify GPCR sequences based on their amino acid composition. The discrimination power of machine learning techniques, like support vector machines, had also been used to classify GPCRs at different levels [17]. GPCRPred [18] implemented SVM to classify GPCR sequences based on their dipeptide composition. There are other methods that are implemented on the transmembrane pattern analysis as these regions are known to be conserved across GPCRs of similar functionality [19]. Hidden Markov models have also been utilized to predict and classify GPCR sequences [20,21]. A majority of these methods use the amino acid composition or the dipeptide composition for classification, or both [22]. A recent method PCA GPCR [23], has used a comprehensive set of 1497 sequence derived features to classify GPCRs into different levels.

In the present study, we have also made an attempt to compare the accuracy of this new version of HMM-ModE to the existing methods available for the classification of GPCRs. We have used the dataset used by the method PCA-GPCR [23] for the comparative analysis.

## Results and discussion

### Benchmarking of the method using HMMER2 and HMMER3

An immediate concern in the implementation of the HMM-ModE protocol with HMMER3 is that this version has only local-local alignments. HMM-ModE can improve signals normally associated with substrate specificity which are differentially conserved in protein superfamilies, and should implicitly benefit from global or “glocal” (align a complete model to a subsequence of the target) alignments.

**Figure 1:**
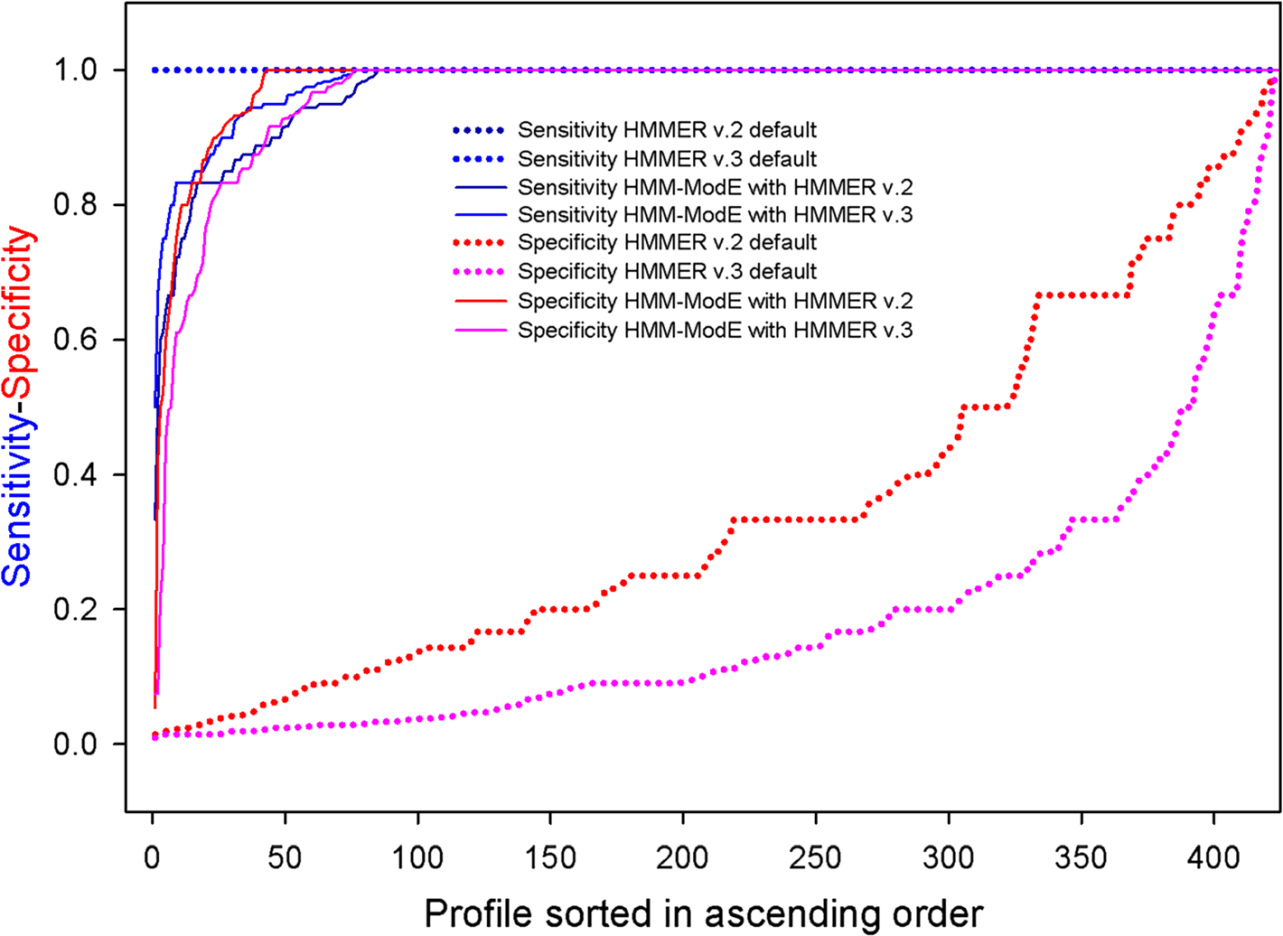
**Benchmarking the HMM-ModE protocol with HMMERv2 and HMMERv3.** The protocol was benchmarked with both versions of HMMER v2 – which permits Glocal alignments – and HMMER v3 which only has local-local alignments on a dataset of 416 enzyme families. Specificity values reported during profile building are improved in all cases, with minimal loss in sensitivity. The specificity improvement is lower for HMMER v3 compared to the earlier version.

In order to benchmark the difference, we have compared the performance of our method using HMMER2 and HMMER3. The HMM-ModE protocol has earlier been applied for large scale annotation of enzymes where the profiles were classified into three classes, based on the number of curated sequences present in the training data set, which were used to assign confidence in the profile [24]. A line diagram showing the performance of both the versions along with default HMMER is shown in Figure 1. A large number of profiles show the expected improvement in specificity using both HMMER2 and HMMER3, without any loss in sensitivity, with over 82.31% and 90.09% reaching a specificity of 1 respectively. There were 15 profiles - 2.1.1.114, 1.3.1.20, 3.6.3.30, 3.1.1.8, 1.12.2.1, 3.4.21.73, 1.14.16.1, 1.2.7.7, 2.5.1.30, 5.5.1.1, 1.1.1.149, 2.6.1.85, 4.2.3.19, 2.6.1.45 and 3.2.1.135 - out of total 416 enzymes, which showed improved specificity of greater than 20% with HMMER 2 over HMMER3 and can be considered extreme cases. All of these profiles had discontinuous match states which were clearly benefited in using a global alignment. In addition to the threshold identified using the HMM-ModE protocol, the low specificity reported during profile-building using HMMER3 should serve as a caution in a mining or large scale annotation exercise. Extreme cases where HMMER3 showed improved specificity of greater than 20% over HMMER2 were the 6 profiles - 4.2.1.78, 1.14.12.11, 1.14.12.3, 3.4.21.20, 1.13.11.39, 4.1.3.38. These were all made up of small numbers of sequences (<10), with continuous match states – beneficial to the local-local alignment. The use of small numbers of training sequences is not recommended in the protocol, as n-fold cross-validation used to determine the threshold further reduces the positive set in each trial. A pre-processing step of clustering sequences to segregate different folds is part of the protocol which efficiently separates isozymes and subunits from multimeric proteins which are classified under the same function. This step also allows for the identification of incorrectly classified proteins in the training set. The sensitivity of the HMM-ModE protocol with HMMER3 is, as expected, better than that of HMMER2, with only six profiles from the dataset falling below 80% against thirteen for the older version. The reduction in sensitivity is seen in sub-families with similar folds and differing substrate specificities, where a false-positive may score higher than a true-positive. As the threshold is optimised on the basis of the highest values from the Mathew’s correlation coefficient, there is a loss in sensitivity in these cases.

The migration of the method to newer version of HMMER has the advantage of higher search speed of execution which is a driving feature in large scale functional annotation. The speed improvements using HMMER3 is evident from a case study, where the HMM-ModE profiles of AGC kinase protein sub families were scanned against the Uniprot database using 'hmmsearch’ from HMMER2 and HMMER3 as shown in the Additional file 1. However, besides conviction in the accuracy and the size of the training dataset, which was the criteria employed with the HMM-ModE protocol used with HMMER2 to assign confidence in the profile, it is recommended that the specificity values reported during profile building be used as an additional criterion in the use of HMM-ModE protocol with HMMER3. Low specificity during profile building will point to cross-specificity with other families in the training set, where the threshold identified using the protocol may not be sufficient to properly discriminate the families during a classification exercise.

### Case study for performance evaluation and validation

The performance of the method is further validated on a set of pre-classified enzyme superfamilies which are clustered according to specific sequence, structure and functional criteria to be used as a gold standard in family and superfamily clustering methods [11]. These include five superfamilies, 91 families, 4,887 sequences and 282 structures and are related proteins with diverged functions proposed to be useful for elucidation of the functions of novel uncharacterized proteins. The training sequences, as shown in Table 1, were selected from each of the five superfamilies. The predictions after the 10 fold cross validation show a significant improvement in specificity over the default HMMER for each of the family as shown in Table 1. The low specificity rates in the default HMM profiles are due to the presence of common fold signal conserved between functionally diverse families. The influence of the fold specific signals is reduced by optimizing the discrimination threshold to separate sequences based on their function. The present method identifies this threshold based on the lowest TP score and the highest FP score. If the highest FP score is greater than lowest TP score then we modify the emission probability of the TP profile using RE score and optimize the threshold using tenfold cross validation, as discussed in our previous paper [9]. Else, if the highest FP score is less than the lowest TP, the defined threshold is much like the gathering scores in PFAM and can classify sequences with maximum sensitivity and specificity. However, if there are no false positives, the default threshold of zero is used.

**Table 1:** **Performance evaluation of the new version of our method HMM-ModE on ‘gold standard’ dataset and comparison with HMM-ModE/HMMER2 **[9]

**SuperFamily**	**Family**	**# Seq**	**HMM-d**		**HMM-ModE with HMMER3 (HMMER2)**	
			**Se**	**Sp**	**Se**	**Sp**
AminoHydrolase	AMP deaminase	28	1	0.90	1(1)	1(1)
	urease	100	1	0.50	1(1)	1(1)
	D-hydantoinase	10	1	0.05	1(1)	1(1)
	Dihydroorotase 2	13	1	0.14	1(1)	1(1)
	Guanine deaminase	11	1	0.22	1(1)	1(1)
	Adenosine deaminase	10	1	0.35	1(1)	1(1)
Enolase	Enolase	215	1	0.88	1(1)	1(1)
	Glucarate dehydratase	26	1	0.20	1(1)	1(1)
	Muconate cycloisomerase	14	1	0.09	1(1)	1(1)
	Chloromuconate cycloisomerase	10	1	0.05	1(1)	1(1)
Crotonase	Enoyl-CoA hydratase	54	1	0.23	1(1)	1(1)
	Histone acetyltransferase	11	11	0.09	1(1)	1(1)
Haloacid Dehydrogenase	P-type atpase	91	1	0.77	1(1)	1(1)
Vicinal Oxygen chelate	Catechol 2,3-dioxygenase	32	1	0.28	1(1)	0.88 (0.88)
	4-Hydroxyphenylpyruvate dioxygenase	26	1	0.60	1(1)	1(1)
	2,3-Dihydroxybiphenyl dioxygenase	23	1	0.20	0.95 (1)	0.71(0.63)
	Glyoxalase 1	12	1	0.20	1(1)	1(1)

HMM-d stands for default HMMER and # Seq stands for number of sequences in each family. Se is Sensitivity and Sp is Specificity. Sn is the sensitivity value calculated as TP/(TP + FN), and Sp is the specificity calculated as TP/(TP + FP). As shown here, there is a significant improvement in the specificity of the predictions with small compromise in the respective sensitivity using HMM-ModE.

The corresponding values of Se and Sp using HMM-ModE with HMMER2 is written in parenthesis.

It is observed, from Table 1, that the specificity increased in four of the five superfamilies tested - the Amino Hydrolase, Enolase, Crotonase and Haloacid Dehydrogenase superfamilies. In most cases there was sufficient difference in the scores of the positive training sequences and the sequences from other classes to calculate a discriminating threshold which provided a specificity of 1.0 with the test data. In the case of Enoyl-CoA hydratase, there were sufficient common match states between related sub-families to provide a case study where the HMM-ModE protocol could improve discrimination by separating positive and negative sequences through dampening from match states differentially conserved between sub-families. The case of the Vicinal Oxygen chelate superfamily provides two cases, 2,3-Dihydroxybiphenyl dioxygenase and Catechol 2,3-dioxygenase where a larger number of negative training sequences score higher than members of the sub-family used as positive training sequences. In these cases, there will be a trade-off between the sensitivity and specificity, as no perfect discrimination can be made in classifying these sequences. However, in general, the present findings lead us to conclude that HMM-ModE tends to reduce the number of false positives without significantly affecting the true positive sequences for their classification into fold (superfamily) and function (family) respectively. These results are significant on this new dataset and complement our previous findings on AGC kinase and GPCR datasets [9].

### Application on GPCR datasets

The GPCR profile HMMs generated, as discussed in Methods, are combined to make a profile database that serve as a resource for classifying novel GPCR sequences at sub family level. Each of these profiles is provided with a discrimination threshold generated during cross validation (See Methods). We have used different datasets (available as Additional file 2), to evaluate and compare our method with the existing ones as described in the following subsections. In addition we have also used the previous version of the method in order to show that both the versions perform equally well but the newer version offers more speed due to the inclusion of very fast and accurate hmm searches.

#### ***D167 dataset***

We have scanned all the sequences from D167 dataset using “hmmscan” from HMMER package. The threshold separating TPs and FPs calculated from HMM-ModE is used as the gathering threshold. The “--cut-ga” option is utilized while scanning the query sequence files with our profile database to make sure that only significant hits are reported which are above this threshold. The human readable output from ‘hmmscan’ is parsed to identify the TPs for the determination of accuracy of our method. These values are then compared with the accuracy values from existing methods and are tabulated in Table 2.

**Table 2:** **The table shows the comparison of our method with other methods and with HMM-ModE/HMMER2 **[9]** to classify D167 dataset**

**Dataset**	**Sub-family**	**Total**	**Predicted**				**Accuracy**				
				**This paper**	[9]	[23]	[15]	[16]	[18]	[22]	[25]
**D167**	Acetylcholine	31	29	**93.54**	**93.54**	100	67.74	90.3	93.6	93.3	96.7
	Adrenoceptor	44	44	**100**	**81.81**	100	88.64	86.4	100	100	100
	Dopamine	38	37	**97.36**	**97.36**	94.74	81.58	78.9	92.1	94.7	92.1
	Serotonin	54	54	**100**	**81.48**	98.15	88.89	79.6	98.2	100	100

Accuracy is calculated as (TP + TN)/(TP + FN + TN + FP) here and throughout.

“Total” is the number of sequences observed in a sub-family, “Predicted” is the number of sequences correctly predicted by our method. The accuracy values for the other methods are directly taken from their articles. The data in bold depicts the results using HMM-ModE with HMMER3 and HMMER2.

The D167 dataset have sequences belonging to Acetylcholine, Adrenoceptors, dopamine and Serotonin sub families from Amine family of Class A Rhodopsin like GPCR. Except for Acetylcholine, the performance of our method is better or equally good for each of these subfamilies. However it is pertinent to note that this is further improved after verification of the sequences in the dataset. Two out of the 31 Acetylcholine sequences not identified as true positive by our method, were found to belong to the histamine subfamiliy from GPCRDB. As the dataset was not annotated with the original accession number of the sequences, we retrieved this information from UniprotKB. The results infer these to have the same Q9QYN8 accession (HRH3_RAT) which is a reviewed entry in UniprotKB annotated as Histamine H3 receptor (*Rattus norvegicus*) and are the same sequence. The effective number of sequences in the Acetylcholine set, therefore, would be 29 all of which are classified accurately by our method giving 100% accuracy for this sub family as well. This also indicates that the use of modified profile HMMs for the classification of GPCR sequences aid in identification of misclassified sequences as well.

#### ***D566 dataset***

The D566 dataset have subfamilies Adrenoceptor, Chemokine, Dopamine, Neuropeptide, Olfactory, Rhodopsin and Serotonin. Except for Chemokine, the accuracy is better for our method using this dataset as well as shown in Table 3. As with the earlier dataset, there is one sequence among the Adrenoceptor sequences that is not picked up by the corresponding profile, and this sequence is probably incorrectly classified, being listed as Dopamine in GPCRDB. The HMM-ModE profiles classify this sequence as belonging to the Dopamine subfamily which infers 100% accuracy for this subfamily instead of 98.48%. The UniprotKB accession of this sequence is O96716 (O96716_BRALA), an unreviewed entry with Dopamine D1/beta receptor annotation. In case of Chemokine subfamily, of the 92 sequences, three are classified into subfamilies other than Chemokine as per GPCRDB classification. These have the accession O75388 (GPR32_HUMAN) a reviewed protein with annotation probable G-Protein Coupled Receptor 32, Q9Z2J6 reviewed protein with annotation Prostaglandin (PD2R2_MOUSE) a D2 receptor/GPCR44 and O88416 (GPR33_MOUSE) a reviewed protein with annotation probable G-Protein Coupled Receptor 32. The number of effective sequences belonging to Chemokine sub family in the D566 dataset is therefore 89, and the correctly predicted sequences by our method are 84 which increases the accuracy to 94.38% from 91.30% for this subfamily. This increase however is slightly lower than the value reported by PCA-GPCR for Chemokine subfamily.

**Table 3:** **The table shows the comparison of our method with PCA-GPCR and with HMM-ModE/HMMER2 **[9]** to classify D566 dataset**

**Dataset**	**Sub-family**	**Total**	**Predicted**	**Accuracy**		
				**This Paper**	[9]	[23]
D566	Adrenoceptor	66	65	**98.48**	**84.84**	98.48
	Chemokine	92	84	**91.30**	**91.30**	97.83
	Dopamine	43	41	**95.34**	**88.37**	93.02
	Neuropeptide	31	31	**100**	**96.77**	96.77
	Olfactory	84	84	**100**	**100**	100
	Rhodopsin	183	183	**100**	**100**	98.36
	Serotonin	67	67	**100**	**77.61**	97.01

“Total” is the number of sequences observed in a sub-family, “Predicted” is the number of sequences correctly predicted by our method. The accuracy values for the other method are directly taken from their article.

The data in bold depicts the results using HMM-ModE with HMMER3 and HMMER2.

#### ***D1238 and D365 dataset***

The D1238 dataset have sequences belonging to Rhodopsin like, Secretin like and Metabotropic/glutamate/pheromone like GPCRs while the D365 dataset have sequences belonging to Rhodopsin like, Secretin like and Metabotropic/glutamate/pheromone like GPCRs, Fungal Pheromone, CAMP receptor and Frizzled/Smoothened GPCRs. Table 4 shows the result after scanning the sequences from both these families. The accuracy for the each of the families in these datasets is lower than that predicted by PCA-GPCR except for CAMP, Frizzled/smoothened and Fungal Pheromone receptors. The accuracy values for these families are reported to be 100%. The lower accuracy for the Secretin and Metabotropic/glutamate/pheromone family for both D1238 and D365 dataset is due to lack of well characterized sequences that were available for training. Rhodopsin on the other hand is the largest family of GPCRs but data used for training from Uniprot does not contain a reasonable number of sequences for certain subfamily level (like Prostacyclin, Thromboxane, Neurotensin, GPR-12, GPR- 20 and others). It is expected that as more well characterized sequences populate these subfamilies, the profiles built will increase their accuracy at family level.

**Table 4:** **Comparison with PCA-GPCR and HMM-ModE/HMMER2 **[9]** to classify D1238 and D365 dataset**

**Dataset**	**Sub-family**	**Total**	**Predicted**	**Accuracy**		
				**This paper**	[9]	[23]
D1238	Rhodopsin like	1103	1030	**93.38**	**95.10**	99.91
	Secretin like	84	66	**78.57**	**78.57**	98.81
	Metabotropic/glutamate/pheromone	51	47	**92.15**	**60.78**	98.04
D365	Rhodopsin like	232	200	**86.20**	**87.50**	95.69
	Secretin like	39	23	**58.97**	**58.97**	87.18
	Metabotropic/glutamate/pheromone	44	34	**77.27**	**54.54**	88.64
	Fungal Pheromone	23	23	**100**	**78.26**	95.65
	CAMP receptors	10	10	**100**	**100**	100
	Frizzled/smoothened	17	17	**100**	**100**	64.71

The accuracy values for the method PCA-GPCR are directly taken from their article. The data in bold depicts the results using HMM-ModE with HMMER3 and HMMER2.

#### ***GPCR_human dataset***

Similarly, in order to assess the performance of our method another data from GPCRDB, which populates the sequences in the corresponding family and subfamilies by mining sequences from NCBI’s NR database using profile HMMs, was used. The dataset is named as GPCR_human and was used to compare the performance of our method against PCA-GPCR as shown in Table 5. The results show the performance of our method in terms of accuracy for a set of subfamilies from GPCR_human dataset. We ran PCA-GPCR on the same dataset for comparing the performance with the proposed method. It is observed here that both the methods work equally well for Acetylcholine, Adrenoceptors and Dopamine subfamily sequences except for a slight decrease in specificity for Adrenoceptors for HMM-ModE. On the other hand, for Histamine and Serotonin, the proposed method shows accuracy of 100% and 96.15%, whereas PCA-GPCR reaches only upto 75.00% and 76.92% respectively. In case of Trace amine subfamily, our method classifies 14 sequences out of the total 23 sequences leaving behind 9 sequences unclassified. PCA-GPCR, on the other hand, classifies 18 of the 23 sequences correctly making the sensitivity 78.26 which is better than our method. However, our method does not misclassify the remaining 9 sequences into any other subfamily while PCA-GPCR classifies the 4 out of 5 sequences into other subfamilies (Adenosine, Somatostatin, Gonadotropin type 1 and Tachykinin) rather than trace amine.

**Table 5:** **This table shows the performance of our method, HMM-ModE/HMMER2 **[9]** and PCA_GPCR on GPCR_human dataset**

**Sub-family**	**Total**	**Predicted**	**Accuracy**		
			**This paper**	[9]	[23]
Muscarinic acetylcholine	11	11	**100**	**100**	100
Adrenocceptors	24	23	**95.83**	**95.83**	95.83
Dopamine	17	16	**94.11**	**94.11**	94.11
Histamine	16	16	**100**	**100**	75.00
Serotonin	26	25	**96.15**	**96.15**	76.92
Trace amine	23	14	**60.87**	**60.87**	78.26

“Total” is the number of sequences present in a sub-family. The data in bold depicts the results using HMM-ModE with HMMER3 and HMMER2.

## Conclusions

We have upgraded the HMM-ModE method to use HMMER3 and shown its importance to functionally annotate sequences belonging to hierarchically classified data. In general, the use of only two classes, positive and negative in training, reduces all sequences not belonging to the positive class into the negative class. With large datasets, the negative training probabilities would tend to be the same as the null probabilities. As negative training data is significantly larger in size than positive training data, the speed of implementation of the method HMM-ModE improves by only selecting false positives from the negative training data, thus limiting its size to those sequences that significantly influence discrimination. The protocol has now been implemented for use with HMMER3, which will permit large scale sequence classification projects through its improved speed. However, besides curated and sufficiently large training sequence datasets, it is recommended that the specificity reported during training be used as a caution in assigning confidence to a profile in such an exercise.

**Figure 2:**
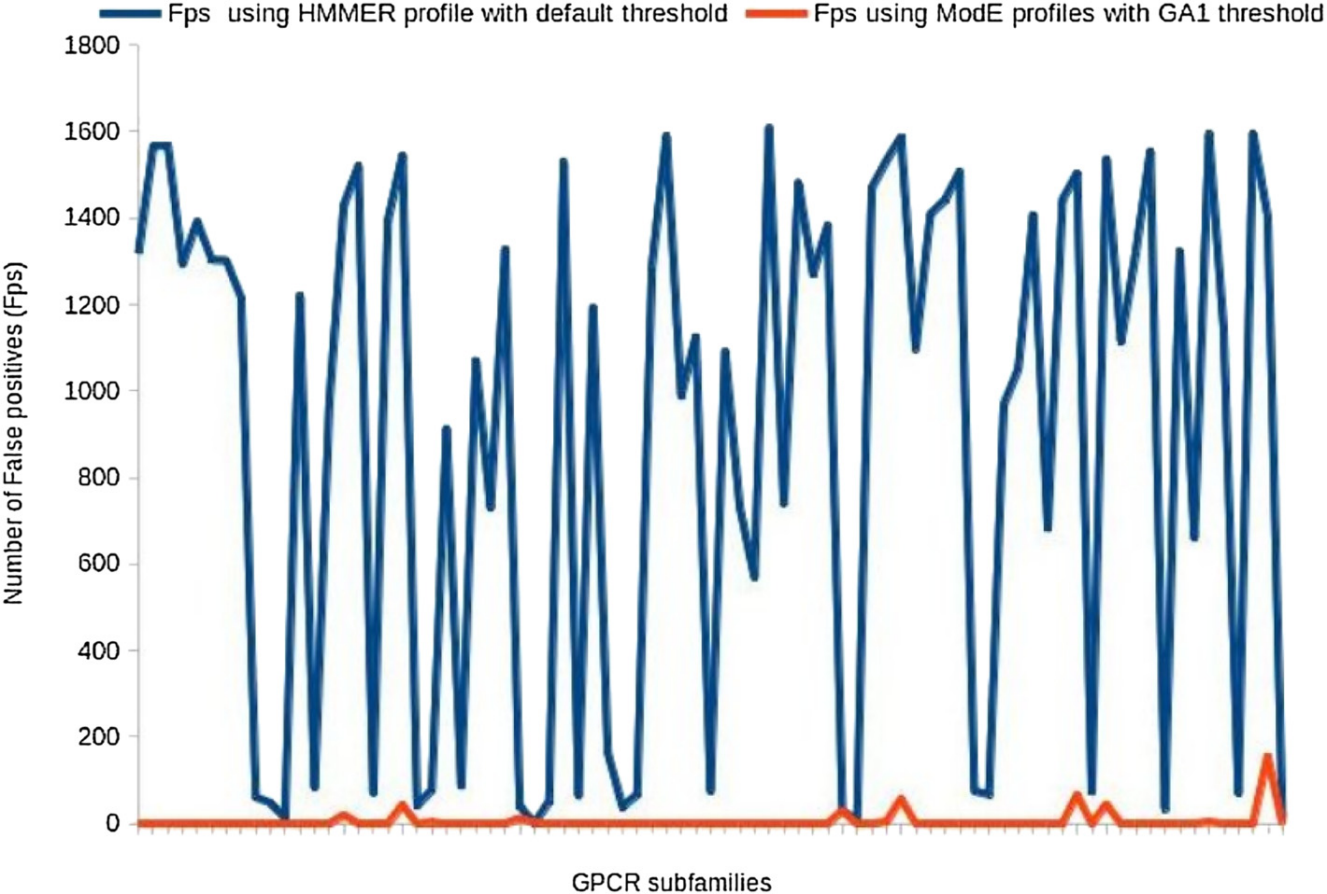
**Comparison of number of false positives while using HMMER and HMM-ModE.** The line diagram in this figure shows a comparison of number of false positives picked up by each of the GPCR subfamily profiles using HMMER profiles with default threshold and the HMM-ModE profiles with a discriminating threshold (similar to the GA1 threshold used by Pfam) generated through 10-fold cross validation as discussed in our published work [9]. As shown in the figure, there is a remarkable reduction in the number of false positives when our method is used which is helpful in the annotation of protein sequences with high specificity.

Using the GPCR dataset we also conclude that the use of sequence profiles, which are built to discriminate between fold and function specific signals, instead of using sequence features like amino acid or di-peptide composition for classification of GPCR sequences at subfamily level is important for accurate functional annotation. The study has been validated and found to be most accurate on datasets, D167 and D566, compared with the earlier published methods. HMM-ModE also performs better as a classifier, when tested on sequences belonging to *Homo sapiens* using the GPCR_human dataset. In the present study, we have shown the use of profile HMMs as an important resource for accurate classification of GPCR sequences at subfamily level. These profile HMMs have modified emission probabilities thus making them highly specific amplified by its direct application on sequences, without the requirement of pre-processing to extract features. Since there is still much to be done to tackle the complicated problem of *in-silico* GPCR classification [26], it is essential to benchmark the algorithms with several datasets in order to maintain a trade-off between the accuracy of the predictions and the comprehensibility of the results. It is not trivial to come up with an algorithm that takes care of classification at several levels, though certain methods are proposed [23]. One of the problems with GPCR classification is that there are a number of shared features across multiple subfamilies, due to the common fold. This is partially alleviated by increasing the number of features – as in PCA-GPCR, which can be used to train machine learning methods.In the case of sequence profiles separating common signals at the level of emission probabilities in Hidden Markov Models, and the use of appropriate scoring thresholds works equally well. The improvement in specificity, in terms of lesser number of false positives, over the use of default profile Hidden Markov Models is seen in Figure 2. It shows a remarkable reduction in number of false positives for each of the GPCR profiles constructed using HMM-ModE over the default HMMER profiles. In general, the present method serve as a useful resource for homology based functional annotation to classify novel protein sequences.

## Methods

**Figure 3:**
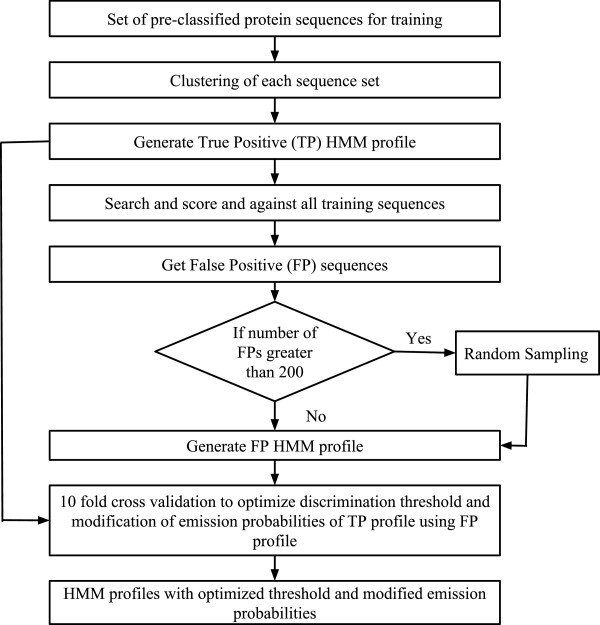
**Schematic flow of how HMM-ModE works on a set of pre-classified protein family sequences.** The figure shows how the method HMM-ModE works, a set of pre-classified sequences (functionally classified) are used which are clustered using MCL in order to obtain clusters of similar sequences. These clusters are aligned separately and HMM profiles are built using ‘hmmbuild’ from HMMER package, these are known as true positive (TP) profiles. The TP profiles are scanned against all the sequences, ideally the profile should pick sequences belonging to the same family but it always picks up sequences belonging to other families as well due to fold specific signals shared across families. We call these as false positive (FP) sequences and generate FP HMM profiles from them. If the number of FPs is greater than 200 then we perform random sampling and then pick a representative set of 200 sequences to generate the FP profile. Both the TP and FP profiles are then aligned using profile-profile alignment from MUSCLE and this alignment is then used to identify the discriminating residues and modify the corresponding emission probabilty of the TP profile. A 10-fold cross validation is also done to identify a discriminating threshold and we use the modified profiles, known as the HMM-ModE profiles, with modified emission probability and the defined discriminating threshold.

The HMM-ModE protocol is now modified to be compatible with HMMER3. The software that implements the protocol is written in a modular fashion that enable the use of best of breed programs for multiple alignments, sequence clustering and HMM profile building and searching. The method uses BLAST [1] for performing all-against-all sequence comparisons and subsequently the Markov Cluster algorithm (MCL) (http://micans.org/mcl/lit/svdthesis.pdf.gz) for clustering of sequences, along with MUSCLE [27] to align the sequences and also for profile-profile alignment of the positive and negative training sequences. HMMER3 [28] has been used to generate profile HMMs and for searching the modified profiles against sequence databases for functional annotation. The workflow for building profiles is outlined as shown in the Figure 3. The scripts and modules to run the method along with a readme file and a test data of protein kinase is provided as Additional file 3.

### Dataset for benchmarking the method HMM-ModE with HMMER3

The hierarchically classified dataset of ENZYME database is used for the comparison of the present version of HMM-ModE. We have used 19Jan2010 and 19Feb2014 release of ENZYME database. The HMM-ModE profiles of 19Jan2010 were built using the in-house method ModEnzA [24], which is an implementation of HMM-ModE with HMMER2 for accurate identification of enzymes. In order to benchmark the method using both versions of HMMER, the dataset was filtered to only include enzyme classes which (i) did not have any change in their size between the two ENZYME database releases (ii) did not contain any fragment sequences and (iii) where the sensitivity was 1 using both default HMMER2 and HMMER3. The 416 enzymes that meet with the above criteria is listed in Additional file 4. Profiles for these enzyme sequences were rebuilt with HMMER3 using the 19Jan2010 ENZYME dataset for direct comparison.

To further validate the discriminating power of the HMM-ModE protocol, a gold standard dataset previously described [11] was pruned to include only families with more than 10 sequences and remove silver standard sequences which were also included in the file. This modified dataset is provided as Additional file 5.

### Dataset to construct GPCR profiles using HMM-ModE

A set of labelled GPCR sequences were downloaded from http://www.uniprot.org/docs/7tmrlist that are classified based on receptor ligand relationship. The total number of sequences in the release 2012_05 of 16-May-2012 is 3071. In order to maintain only a set of well characterized sequences for constructing the profiles, we have reduced the dataset by applying a couple of filters. Firstly, the sequences that are putative, hypothetical or predictive are removed from the dataset. Secondly, only the sequences with seven transmembranes were kept for further analysis and the presence of these seven-transmembrane helices in the reduced dataset was confirmed by GPCR-HMM [29]. Thereafter, we have used 2110 sequences, which belong to different GPCR subfamilies and each of these sub families contain at least three sequences. A table having a list of subfamilies taken for training purpose along with number of sequences in each of these subfamilies is provided as Additional file 6. In cases, where the number of sequences in a subfamily is small (~10 or less), it is advised to mine similar sequences using BLAST [1] and then create the HMM profile using our method for annotation tasks.

### Dataset for comparative analysis

We have used D167 and D566 dataset from one of the recent method, PCA-GPCR, for classification of GPCR sequences which consist of subfamilies classified on the basis of substrate specificity. We have also used two other datasets D1238 and D365, from the same resource which contain sequences at the class level having a broader functional classification. A new dataset, GPCR_human, have been created from the GPCRDB database [13] to test the respective methods. This dataset contains sequences belonging to Homo sapiens and includes Muscarinic Acetylcholine, Adrenoceptors, Dopamine, Histamine, Serotonin and Trace amine subfamilies having 11, 24, 17, 16, 26 and 23 sequences respectively.

### Procedure to construct HMM-ModE profiles of GPCR proteins

In order to generate highly specific profile HMMs from the curated GPCR dataset, the sequences of each subfamily were aligned separately using Praline-TM [30]. The subsequent alignments of each subfamily were combined to create a master alignment using MAFFT [31] profile-profile alignment. For each sub-family, a HMM profile is generated from the alignment of its sequences which is then used to identify the false positive sequences from rest of the sub-families. The True Positives (TPs) are defined as the members belonging to a particular subfamily while the sequences belonging to different subfamilies which are picked up by the TP subfamily when scanned across all the sequences, are categorized as False Positives (FPs). Having known the TPs and FPs, the master alignment is then used to retrieve the TP and FP alignments. The purpose of using the master alignment is to ensure that the multiple alignment columns are comparable between the corresponding HMM match states for the true and false positive profiles. This is a critical requirement, as the emission probabilities from the corresponding columns are extracted directly from the HMM profile for calculation of relative entropy, and the resultant modification of emission probability. The TP and the FP alignment are subsequently used to identify the discriminating positions for fold specific signals. The emission probabilities corresponding to these positions are modified using relative entropy as discussed in our earlier work [9]. The profiles modified in terms of changed emission probabilities are used with a discrimination threshold, which is the value for the threshold used in profile HMM built from HMMER3 [28], generated through tenfold cross validation. The use of this cut off enables the profile to make highly specific classification at a finer functional level like subfamily. These profiles along with the discrimination threshold are made available as Additional file 7.

### Availability of supporting data

The data used in the manuscript is provided as following Additional files.

## Competing interests

The authors declare that they have no competing interests.

## Authors’ contributions

AML guided the work and gave the idea; SS implemented the work and modified the manuscript. Both authors read and approved the final manuscript.

## Supplementary Material

Additional file 1 Figure showing comparison of time elapsed to scan the AGC protein kinase sub families using ‘hmmsearch’ from HMMER2 and HMMER3 against the Uniprot database. Click here for file

Additional file 2 GPCR dataset used for comparative study in this manuscript. Click here for file

Additional file 3 Scripts to run the method along with a test data and a readme file. Click here for file

Additional file 4 List of 416 enzymes used for benchmarking the method using both HMMER2 and HMMER3. Click here for file

Additional file 5 Sequences belonging to the gold standard families used in this study. Click here for file

Additional file 6 List of GPCR subfamilies used along with the number of sequences in each of these. Click here for file

Additional file 7 **Modified Profile HMM database of the GPCRs generated using our method.** It can be directly used to mine sequences using ‘hmmsearch’ with –cut_ga option from the HMMER package. Click here for file
