# Supplementary material for: HMM-ModE: implementation, benchmarking and validation with HMMER3
Source: BMC Res Notes. 2014 Jul 30;7:483. doi: 10.1186/1756-0500-7-483 (PMC4236727; doi:10.1186/1756-0500-7-483)
Supplement: Additional file 1 — Figure showing comparison of time elapsed to scan the AGC protein kinase sub families using ‘hmmsearch’ from HMMER2 and HMMER3 against the Uniprot database. [file 1756-0500-7-483-S1.pdf]

**Additional Figure 1.**

**Figure Title:** Comparison of time elapsed to scan the AGC protein kinase sub families using hmmsearch from HMMER 2.3.2 and HMMER 3.0 against the Uniprot database.

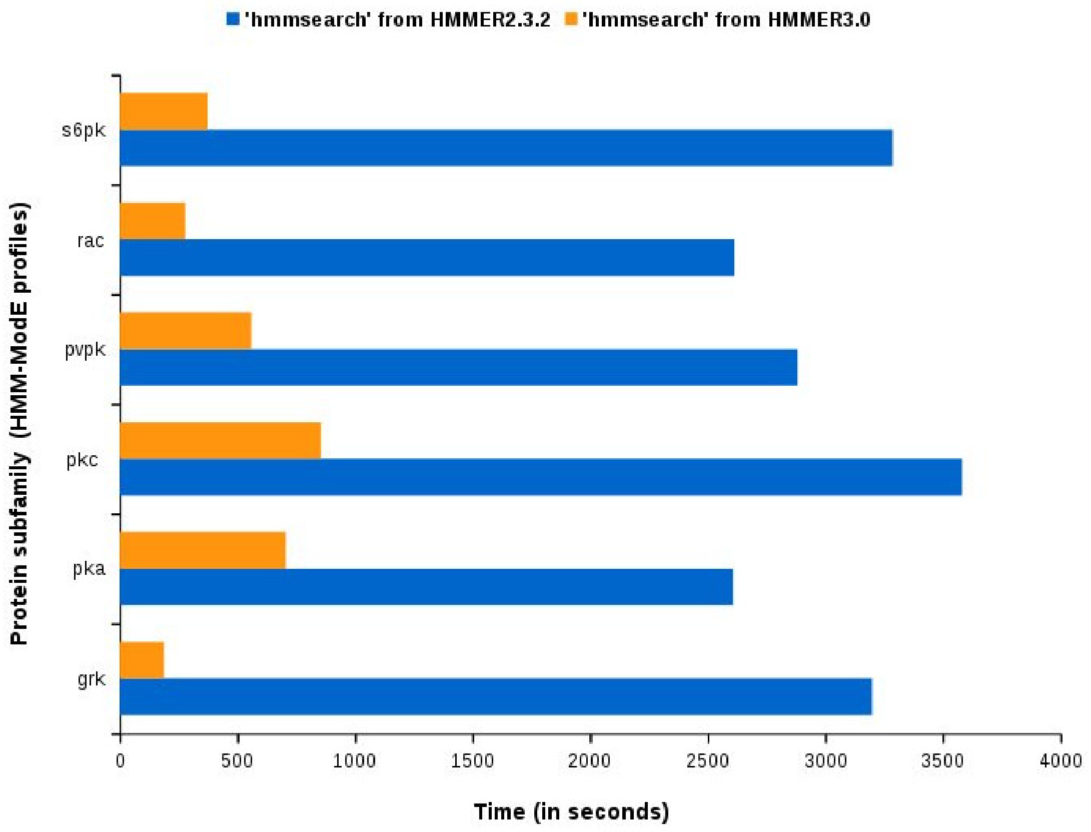


The bar plot shows the time elapsed when the HMM-ModE profiles of AGC protein kinase sub-families are scanned across the Uniprot database having 542503 sequences. The six sub-families used are G protein couples receptor kinase (GRK) having 58 sequences, Cyclic nucleotide regulated protein kinase (PKA) having 69 sequences, Protein kinase C (PKC) having 139 sequences, Flowering plant PVPK! Protein kinase (PVPK1) having 50 sequences, RAC/Akt protein kinase, related to PKA and PKC (RAC) having 23 sequences and Ribosomal protein s6 kinase (S6PK) having 41 sequences. There is a significant reduction in the time required to run the search using 'hmmsearch' from HMMER 3.0. This shows that the inclusion of HMMER 3.0 with HMM-ModE will help to utilize the accelerated HMM searches utility of the newer version of HMMER which in turn will be useful for large scale protein function prediction.
