# Supplementary material for: HMM-ModE: implementation, benchmarking and validation with HMMER3
Source: BMC Res Notes. 2014 Jul 30;7:483. doi: 10.1186/1756-0500-7-483 (PMC4236727; doi:10.1186/1756-0500-7-483)
Supplement: Additional file 6 — List of GPCR subfamilies used along with the number of sequences in each of these. [file 1756-0500-7-483-S6.pdf]

Table 1: GPCR sub-families used for training our method along with number of sequences present.

Sub family	Number of sequences
Acetylcholine Muscarinic	34
Adenosine	29
Adrenergic adrenoceptors	70
Anaphylatoxin	23
Angiotensin	22
ApJ like	9
Bombesin	13
Bradykinin	16
Calcitonin	11
Calcium Sensing	4
Camp	11
Cannabinoids	15
Cd97	3
Chemokiness_Receptor_like	3
Chemokine	76
Cholecystokinin	14
Corticotropin releasing factor	10
Cysteinyl Leukotriene	13
Dopamine	38
Duffy antigen	11
EMR	8
Endothelin	8
Family T2R	165
Fmet-leu-phe	23
Follicle-stimulating-hormone	15
Free fatty acid receptor like	9
Frizzled/Smoothened	78
Fungal Pheromone	12
Gaba-b	6
Galine like	12
Glucagon	6
Gonadotropin releasing hormone	16
Gpcr Bile acid receptor	4
GPR56	6
GPR64	3
Growth hormone secretagogue	6
Histamine	23
Hydroxycarboxylic acid receptor	3
Interleukin8	3
Latrophilin	11
Leukotriene	4
LGR like	6

Lutropin-choriogonadotropic-hormone	7
Lysosphingolipid_LPA	34
mass_protooncogene_mgr	47
Melanin-concentrating_hormone	10
Melanocortins	71
Melanotonin	14
Metabotropic glutamate	27
Methuselah like receptor	5
Neuromedin U	7
Neuropeptide_Y	36
octopamine	10
olfactory	398
Opioid	22
Opsins	194
Orexins	6
Pacap	3
Parathyroid hormone	8
Platelet activating factor	7
Prokineticin	6
Prolactin releasing peptide_GPR10	4
Prostaglandin	10
Proteinase activated like	14
Purinoceptors	21
Secretin	3
Serotonin	80
Somatostatin angiotensin	4
Somatostatin	23
Tachykinin	22
Taste receptor	20
Thyrotropin relasing hormone	6
Thyrotropin	8
Trace Amine	41
Urotensin II	6
Vasoactive intestinal polypeptide	8
Vasopressin oxytocin	31
Viral	15
Vomeronasal receptors	30
